# The Heart Failure “Pandemic” in Japan: Reconstruction of Health Care System in the Highly Aged Society

**DOI:** 10.31662/jmaj.2018-0049

**Published:** 2019-05-16

**Authors:** Mitsuaki Isobe

**Affiliations:** 1 Sakakibara Heart Institute, Tokyo, Japan

**Keywords:** heart failure, aging society, frailty, multidisciplinary approach

## Abstract

An increase in the number of patients with heart failure is an international health-related problem. In advanced countries, the number of such patients has rapidly increased since the beginning of the 21^st^ century, raising an important issue regarding medical practice and public health. In 2010, the concept of “heart failure pandemic” was proposed, and it has been recognized as a global social/economic issue. In particular, the number of elderly patients with heart failure has increased with the rapid aging of society and a decrease in the number of children in Japan. A rapid increase in the number of heart failure patients increases stress and social disease-related/medical economic burdens on individuals and their families. The prognosis of patients with chronic heart failure is unfavorable, and the quality of life markedly reduces. To improve the prognosis of elderly patients with heart failure and reduce the readmission rate, the innovation of a medical-care-providing system for heart failure is required. In addition to the provision of medical practice based on a potent heart failure disease control program, manifold strategies, such as lifestyle improvements, self-care practice, cardiac rehabilitation, and environmental intervention, are essential. It is necessary to innovate hospital-based medical practice to a regional-care-system-based medical care system. In addition, to efficiently promote future heart failure strategies, an investigational study with disease registration must be conducted. Recently a new basic act on countermeasures to cardiovascular diseases has been established which may help the reform for this purpose.

## 1. “Heart failure pandemic”

As indicated by the Rotterdam study, the morbidity rate of heart failure increases with aging ^(1)^. In males, it rapidly increases from 75 to 80 years of age, reaching approximately 6% at 90 years of age. The rapid aging of society is a common phenomenon in the world, and an increase in the incidence of heart failure is a global issue ^(2), (3), (4), (5), (6), (7)^. Internationally, about 26 million adults are living with heart failure ^(6)^. The same trend is emerging in Asia as well ^(8), (9)^. In Japan, the rapid aging of society is the most advanced in the world, and the morbidity rate of heart failure has also rapidly increased. Currently, there may be approximately 1,000,000 patients with chronic heart failure in Japan, although there are no accurate epidemiological data ^(10)^. The number of patients who will newly develop heart failure in 2025 is estimated to be 370,000. Although the total population will decrease from 126 to 110 million persons in 2035, the number of heart failure patients is estimated to reach 1,300,000 ^(11)^.

Geriatric heart failure is characterized by frequent readmission related to acute exacerbation. A cohort study (JCARE-CARD study) in Japan showed that the readmission rates within six months after discharge and after one year in patients with heart failure were 27% and 35%, respectively, and were similar to those reported in Europe and the United States ^(12)^.

“Heart failure pandemic” is a warning that represents the coming of a critical social status related to a rapid society-aging-related increase in the number of heart failure patients, social burdens, and extraordinary health expenditure ^(4), (13), (14), (15)^.

## 2. Changes in the Clinical Features of Heart Failure

The profile of patients with heart failure has markedly changed recently (Table 1). Heart failure treatment has been primarily performed in young patients, and the goal of treatment was to prolong the life span. Typically, these patients do not have commodities. Hospital health care professionals are responsible for their medical care. For treatment, adequate drugs or devices are used based on clinical evidence. Even in the future, this viewpoint cannot be lost, but, currently, the number of elderly patients with various concomitant diseases or weakness is rapidly increasing, and the goal of treatment is to prolong and live a healthier life, avoid readmission, and improve the quality of life (QOL). Furthermore, treatment measures may consist of disease control, such as education, lifestyle guidance, rehabilitation, and palliative care as a final method. A system for a multidisciplinary medical team to support therapeutic strategies selected based on patients’ wishes in the area of their residence is required.

**Table 1.** Changes in the Characteristics of Patients with Chronic Heart Failure/Contents of Medical Services.

	Heart failure previously treated	Geriatric heart failure
Age	Young persons	Elderly persons
Life environment	Employed persons/family	Persons living alone/elderly husbands and wives
Left ventricular function	Decrease in the ejection fraction	Normal ejection fraction
Complication	Absent	Concomitant presence of various diseases/frailty
Cause of readmission	Reduction of exercise tolerance	Insufficient control/other diseases
Category of disease	Chronic disease	Fatal disease
Reason for death	Ejection fraction reduction/congestion/sudden death	Other diseases/systemic weakness
Goal of treatment	Rehabilitation/lifetime prolongation	Health expectancy prolongation/quality of life improvement
Initiative in treatment	Medical side	Patient side
Primary persons responsible for treatment	Physicians/nurses	Multi-occupational/medical team
Place of treatment	Admission	Outpatient clinic/home

Patients with heart failure are repeatedly readmitted, and they have several comorbid disorders; therefore, the condition varies, being complex. Concerning the prognosis, the five-year survival rate is less than 50% ^(16)^. It is lower than in patients with breast cancer or prostatic cancer, and therefore, heart failure must be regarded as a progressive, fatal disease. Despite advances in medicine, the pathogenesis of heart failure and the mechanism of progression to a severe status remain to be clarified. No causal therapy has been established. For this reason, advance care planning (ACP) or palliative care must be considered in patients with heart failure.

**Figure 1.**
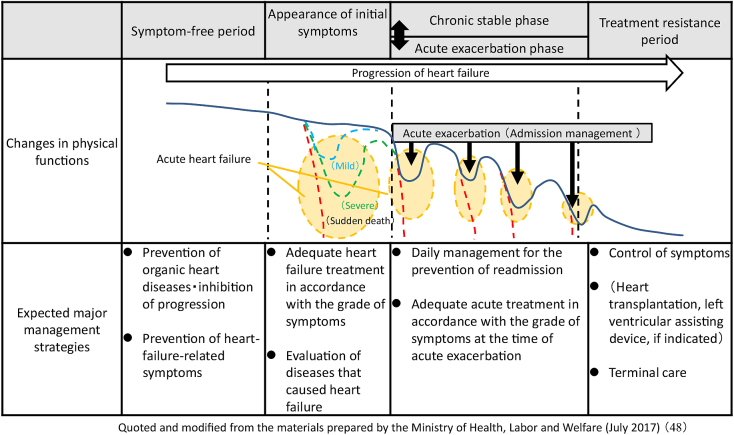
Clinical course and management plan for heart failure ^(48)^.

In Japan, two major diseases that cause chronic heart failure are myocardial infarction and hypertension ^(12)^. Acute-phase coronary reperfusion therapy for acute myocardial infarction, which had been often fatal, has improved the lifesaving rate ^(17)^. Emergent coronary intervention for acute myocardial infarction can be performed in more than 90% of cardiovascular emergent wards in Japan ^(18)^. However, even when lifesaving is achieved, heart dysfunction remains as a sequela in many patients. Subsequently, the process of cardiac remodeling gradually deteriorates the heart function even in the absence of symptoms, leading to heart failure. Subsequently, long-term therapeutic intervention is required, but admission and discharge are repeated every time heart failure exacerbates, resulting in a fatal outcome (Figure 1). Hypertension, valvular disease, and cardiomyopathy also show a similar clinical course.

In the majority of elderly patients with heart failure, contractile dysfunction is absent: heart failure with preserved ejection fraction (HFpEF) ^(19), (20), (21), (22), (23)^. According to the JCARE-CARD study, HFpEF accounted for 26% of 1,692 patients with heart failure, excluding those with valvular disease. In the elderly patients, the rate of HFpEF was higher than in those with heart failure related to contractile dysfunction. As a disease that causes HFpEF, hypertension accounts for 44%, suggesting that the rapid aging of society and hypertension contribute to an increase in the incidence of HFpEF in Japan ^(12)^. The prognosis of patients with HFpEF is unfavorable compared with the prognosis of those with heart failure related to contractile dysfunction, but no effective treatment method has been established.

Fried et al. proposed the entity of frailty in 2000 ^(24), (25)^. A frailty cycle consists of physical, mental, and social fragility. It refers to a condition in which various aging-related functional changes or a reduction in reserve increases frailty to health damage. It must be considered that frailty may be prevented/reduced by adequate support/intervention. Etiological factors for frailty in elderly persons vary: malnutrition, physical hypofunction, sarcopenia-related muscle weakness, a decrease in the opportunity of social interactions, and chronic diseases, including heart failure ^(26), (27), (28), (29)^. Frailty treatment and heart failure management may be the two sides of the same coin.

In addition, geriatric heart failure is characterized by the presence of comorbid disorders in most patients: dementia, cerebral infarction, chronic kidney disease, diabetes mellitus, peripheral vascular occlusion, chronic obstructive pulmonary disease, cancer, depression, and bone and joint diseases ^(30), (31), (32), (33)^. Each patient has several disorders. Polypharmacy for such disorders makes the state of the patient more complex in some cases ^(34), (35)^. The major cause of readmission of elderly patients with heart failure is the inappropriateness of self-care for daily life. The difficulty in achieving behavior change in such patients is another serious problem in the management of the disease. Moreover, the behavior of the medical staff for such patients should be flexible, depending on individual patient as compared with younger patients. In addition to heart failure management, diagnosis and guidance beyond the extent of specialty based on the general condition are necessary ^(31), (36)^.

“Heart failure pandemic” also refers to a state in which a rapid society-aging-related increase in the number of heart failure patients with such complex backgrounds cannot be covered by the current medical basis.

## 3. Social Burdens for “Heart Failure Pandemic”

Japan is one of the top countries in the world showing a very long average life span ^(37)^. However, differences from healthy life expectancy in males and females are about 9 and 12 years, respectively; nursing/support is required for these periods ^(38)^. Nursing is an extremely serious social burden. The primary factors that require nursing include stroke, dementia, an advanced age, and bone and joint diseases in Japan. Heart disease and stroke account for more than 20% of patients requiring nursing. Frailty-based elderly-specific characteristics termed “geriatric syndrome” consist of physiological aging and morbid aging.

The concept of geriatric syndrome is medically and socially important. Recently, the number of families consisting of elderly persons alone and that of elderly persons who live alone have increased, and burdens for nursing in our society are increasing. As of 2016, households involving persons aged ≥65 years account for 48.4% of all households ^(39)^. The rate of persons who live with their children was approximately 70% in 1980, but it decreased to 39.0% in 2015. One-person households or those consisting of a husband and wife accounted for <30% in 1980, but the percentage increased to 56.9% in 2015. Heart failure under such circumstances makes it difficult to select a care facility due to a reduction in the activity level, increase in the burdens for nursing, and delay in decision-making. In addition, there are various problems, including poverty and social support systems ^(40)^. Concerning heart failure, there are only a few special consulting services, differing from cancer. Thus, the limitation of information also promotes social isolation ^(41), (42)^. In many underpopulated areas, the rapid aging of society led to the breakdown of regional communities. As the duration of heart failure is prolonged, problems become more serious and protracted.

## 4. Medical Economic Burdens

Currently, the total annual health expenditure in Japan exceeds 40 trillion yen ($330 billion) and is still increasing ^(43)^. One of the reasons is an increase in the number of elderly persons. A situation in which the working-age population and the number of children decreases while the need for medical care increases has been reached. Recently, effective, costly treatment methods have been innovated. Extremely expensive treatment instruments, such as devices for cardiac resynchronization therapy with defibrillation, catheters for percutaneous aortic valve replacement, stents for vascular treatment, and implantable heart assist devices, are used for heart failure treatment, although this is not frequently discussed in comparison with anticancer drugs. Health expenditure on cardiovascular treatment exceeds 6 trillion yen ($50 billion) per year, which is approximately 1.5 times higher than that for cancer ^(43)^. In addition to the high cost of equipment/devices for treatment, admission-related health care costs raise a financial issue on heart failure treatment with repeated readmission. In Japan, the average admission period in patients with heart failure is ≥2 weeks, and expenditure required for a session of heart failure admission per person is estimated to exceed one million yen ($10 thousand) ^(44), (45)^.

Advances in medical practice, which contribute to patient saving, life prolongation, and an improvement in the QOL, should be welcomed, but the indication of treatment must always be carefully examined, considering medical and financial resources, which are not infinite.

In general, elderly heart failure patients have multiple problems in their heart. Therefore, their medication often includes antihypertensive drugs, statins, β-blockers, renin-angiotensin system blockers, diuretics, direct oral anticoagulants, antiarrhythmic agents, and others. Some of the new drugs for cardiovascular diseases are quite expensive, and elderly patients with heart failure tend to receive many drugs due to comorbidities. Expensive medical charge for outpatients with heart failure could promote decreased adherence to the medication after discharge and result in further deterioration of health economics.

## 5. Heart Failure Treatment-providing System to Be Desired

The most important solution for heart failure pandemic should be primary prevention, as discussed later. Fortunately, the incidence of heart failure can be reduced through the management of atherosclerosis and hypertension. In the future, it may be necessary to advance acute-phase and recovery-expected patient care and establish a heart failure treatment system for better support for the elderly ^(46)^. More appropriate and active acute care of heart failure should be associated with better prognosis, especially in elderly patients. Other important medical progresses to improve or prevent heart failure are less invasive measures to treat structural heart disease and other devices to treat heart failure and arrhythmias.

**Figure 2.**
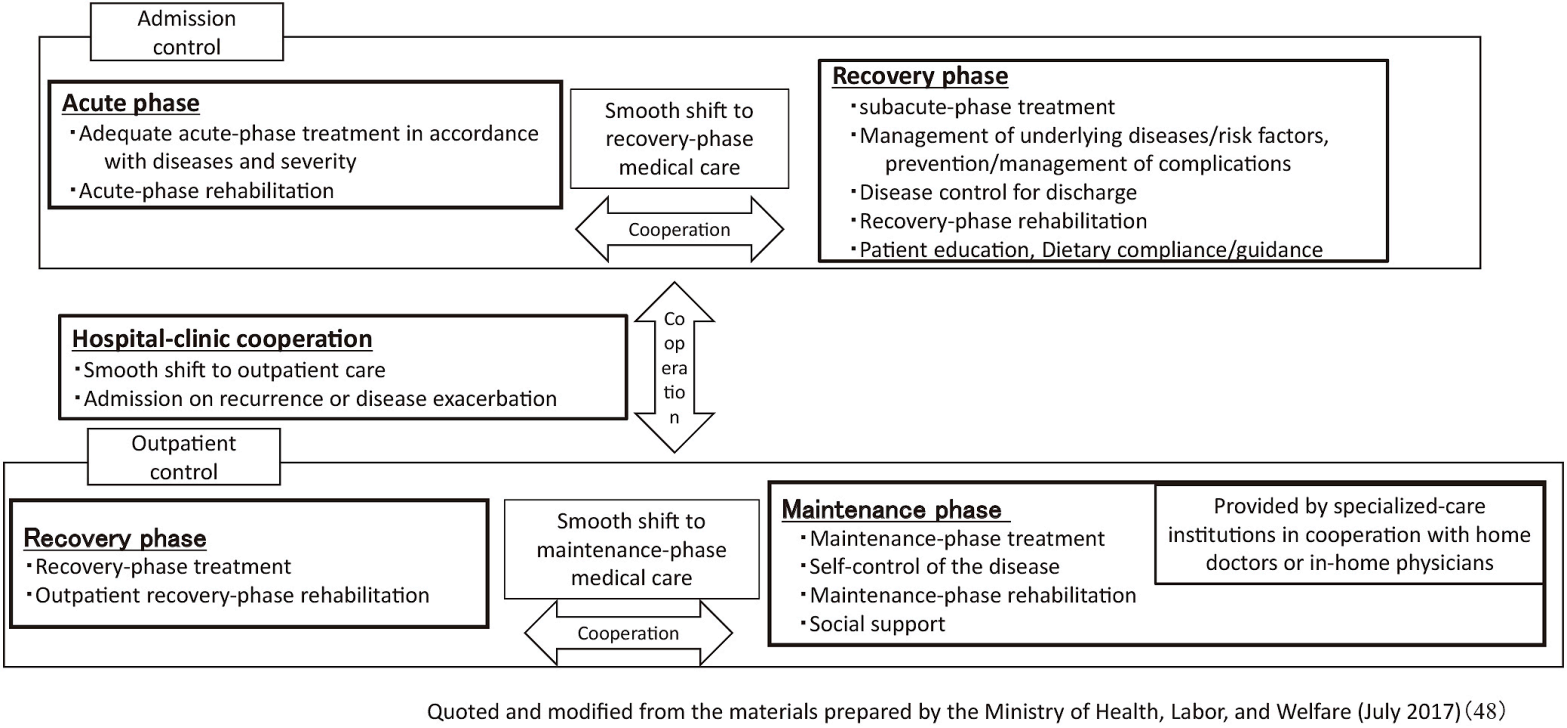
Health care providing system in each phase of heart disease ^(48)^.

In 2016, the 5-year plan of a medical care system was proposed by the Japanese Circulation Society and Japan Stroke Society ^(47)^. In July 2017, a proposal on a medical system to be arranged was announced by the Ministry of Health, Labor, and Welfare ^(48)^. In this proposal, it is emphasized that a new regional medical cooperation system covering the maintenance phase should be established in addition to an acute-phase system to improve the prognosis over a long period from the acute phase and maintain the QOL (Figure 2). Seamless patient flow from acute care facilities to recovery- and chronic-phase care facilities, a multi-occupational team responsible for its management, and patient management based on a disease control manual are proposed.

### A. Rehabilitation

In Japan, rehabilitation has been discussed, considering stroke and orthopedic diseases. In particular, physical therapy has been regarded as playing a central role. However, the goal of rehabilitation for cardiovascular diseases, such as heart failure and myocardial infarction, is to achieve physical function maintenance/improvement by physical therapy and perform comprehensive disease control involving lifestyle/compliance/nutritional guidance, mental care, and social life support for recurrence prevention or prognosis improvement ^(49)^. Rehabilitation for cardiovascular diseases should be started immediately after the onset of heart failure or myocardial infarction and is continued during admission. It aims for early ambulation after admission, functional recovery, and prevention of frailty. Further functional recovery and readmission prevention are targeted through continued outpatient rehabilitation. In addition, for patients who find it difficult to visit a hospital, continuation of rehabilitation at home is recommended ^(50), (51), (52)^.

Exercise tolerance is a prognostic factor for heart failure. Exercise therapy improves exercise tolerance, health-associated QOL, and long-term outcome through manifold effects. Belardinelli et al. reported that exercise therapy decreased the relative risk of cardiac death by 63% in patients with chronic heart failure ^(53)^. Furthermore, the ExTraMATCH study indicated that exercise therapy improved survival and readmission avoidance rates ^(54)^. Provoking factors for readmission in patients with heart failure include environmental factors that can be prevented in addition to medical factors. Multi-occupational intervention, as described in the next section, during outpatient cardiac rehabilitation contributes to more effective disease control ^(55)^. The J-HOMECARE study showed that such a disease control program reduced depression or anxiety, improving the QOL and preventing readmission related to heart failure exacerbation ^(56)^. Therefore, cardiac rehabilitation may be the most effective disease control program including multilateral and comprehensive lifestyle/nutritional guidance, counseling, and patient education in addition to exercise therapy.

### B. Multidisciplinary management

For treatment after the onset of heart failure, the involvement of various occupations is required ^(57), (58), (59)^ (Table 2): nurses responsible for counseling on a disease-associated social life, daily living, drug therapy, consultations, and examinations, dietitians responsible for counseling on diet and nutrition, pharmacists who explain drugs and assist patients to take them, physical therapists responsible for rehabilitation guidance and lifestyle/exercise prescription, clinical technologists who provide laboratory data on the heart to the medical staff and patients, and social workers responsible for counseling regarding post-discharge care/facility reference/counseling and coordination regarding service for long-term care ^(60)^. Concerning physicians, in addition to cardiologists, cardiac surgeons, gastroenterologists, ophthalmologists, dentists, and orthopedists may be involved in accordance with patients’ conditions. In particular, psychiatrists, Liaison nurses, and clinical psychologists must participate in the mental and psychological intervention. Such intervention may improve drug compliance, motor ability, lifestyle, and self-monitoring, preventing weakness, and, thus, facilitating readmission avoidance ^(40), (60)^.

**Table 2.** Multiple Occupations Involved in Heart Failure Treatment.

Physician: Cardiologist, home doctor (practitioner, in-home doctor), cardiac surgeon, ophthalmologist, dentist, orthopedist, psychiatrist
Nurse
Pharmacist
Dietitian
Physical therapist
Clinical technologist
Social worker
Care manager
Person in charge of welfare in a regional administrative area

In addition, even in outpatient care or medical care by home doctors after discharge, multidisciplinary management leads to admission avoidance and shortening of the admission period through the assessment of lifestyle-related background factors, drug compliance, diet, and early detection of slight heart failure exacerbation. Such team-based involvement should be started immediately after admission, and continued during outpatient care, regional care by practitioners, or home care. For this purpose, patient notebooks, combination paths, and checklists are used as communication tools.

### C. Effects of a patient management program

In Europe and the United States, it has long been recognized that such a comprehensive disease control program by a multidisciplinary team prevents readmission related to heart failure, improving the prognosis ^(61), (62)^. A study investigated 600 medical institutions in the United States, and indicated the following heart failure-related readmission-preventing factors: 1. cooperation with regional physicians, 2. strategy-sharing with other hospitals, 3. adjustment of drug therapy plans by nurses on discharge, 4. preparation of an outpatient consultation plan after discharge, 5. provision of information on discharge to home doctors, and 6. sharing of laboratory data on discharge with patients ^(63)^.

Recently, the importance of such an attempt has also been recognized in Japan. In Tottori University, admission care involving the multidisciplinary education of inpatients with heart failure, conferences, optimization of treatment, and rehabilitation through intervention by a multidisciplinary heart failure team markedly decreased the heart failure-related admission and mortality rates in comparison with the period during which standard treatment had been performed ^(64)^.

### D. Community-based integrated care system and cooperation with home doctors

**Figure 3.**
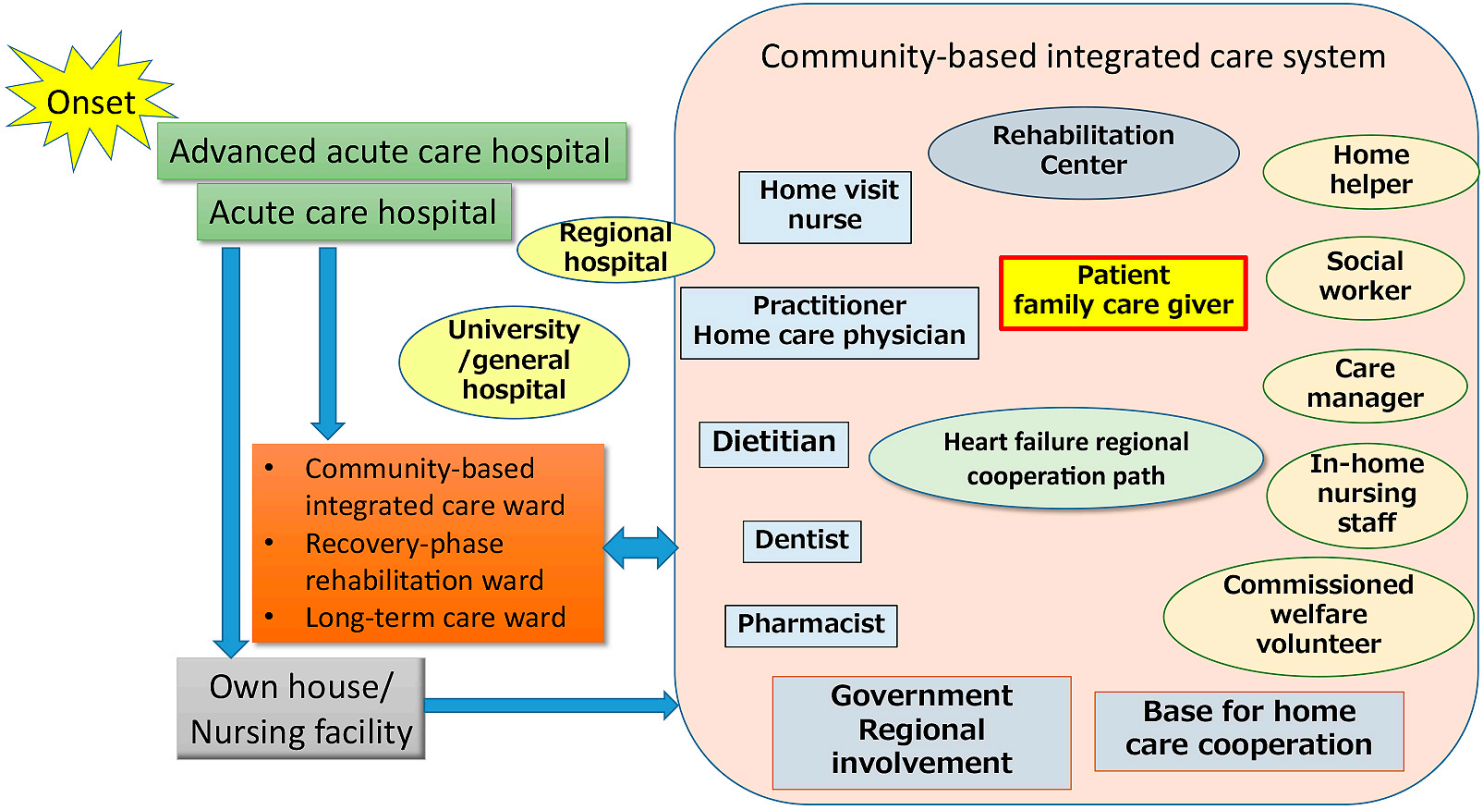
Cooperation of hospitals and community-based integrated care system for heart failure patients.

Patients with heart failure live at home for the longest time after discharge ^(59)^. In modern times, with the rapid aging of society, there are many patients who live alone or care for elderly husbands or wives, and the place of living has been shifting from the home to various facilities: homes for the aged, housings of the aged with various services, rehabilitation facilities for the elderly, special nursing homes for the aged, and group homes. Various service-providing organizations, such as home-visit rehabilitation/nursing offices (care managers) and in-home nursing offices (home-helpers), are involved. In particular, the Government of Japan has promoted community-based integrated care system (Figure 3). It is a policy to support the elderly with reduced living activities and health in a regional community system. Basically, to care for the elderly in the place of living, such as their houses, medical institutions, such as practitioners, in-home doctors, recovery-phase rehabilitation hospitals, and general hospitals, and supporting organizations, involving in-home nursing, home-visit nursing, home-visit rehabilitation, and facility nursing, support them. In this system, care managers primarily care for patients through inter-occupational cooperation.

In a community-based integrated care system, practitioners and in-home doctors, as regional home doctors, primarily support elderly patients with heart failure ^(65)^. In the future, regional heart failure management by home doctors, assistance, and standardization may be necessary ^(66)^. In this situation, the role of hospitals will change. When treating elderly patients with heart failure, it is necessary to prevent weakness and target home return rather than cure. Careful prescriptions and dementia-supposed ward management must be considered for prevention of unnecessary prolongation of admission period.

Concerning hospital-clinic cooperation, it is necessary to aim at horizontal-type cooperation characterized by an equal relationship between home doctors and hospitals, from hospital-based vertical-type cooperation. Hospital-clinic cooperation has been promoted to smoothly conduct referral from core hospitals to home doctors, reverse referral, and hospital transfer. However, for the future cooperative management of rapidly increasing elderly patients with heart failure, it is necessary to share information on individual patients more closely. Multi-job, inter-institutional information sharing through heart failure regional cooperative conferences involving a hospital team and home doctors is ideal. For the prevention of post-discharge recurrence and early detection of deterioration, close communications with visiting nurses are also necessary.

For regional patient information sharing, it may be necessary to standardize charts using the cloud under accurate security control and arrange patient paths/notebooks on heart failure ^(58)^. Various telemedicine devices that are being developed may be useful for collecting patient information, and home doctors who comprehensively utilize them are desirable ^(67)^. Patients may repeatedly move from a hospital to their home, from their home to their child’s family, and to a nursing facility. It is difficult to continue care by activities in a single area alone. Therefore, information-sharing tools covering a broader extent are required, and it is necessary to improve/support the degree of recognition for the establishment of a new system by society as a whole.

## 6. Establishment of a Terminal Care System

Geriatric heart failure leads to death. How patients live in the terminal phase of a refractory disease is an important issue (Figure 1). Currently, various topics on terminal care, such as living wills, palliative care, caregiving, death with dignity, and euthanasia, are discussed. However, there are various issues on terminal care for heart failure ^(68), (69)^. In particular, Japanese people’s view of life and death markedly differs from that of Europeans and Americans; the entity of ACP has not commonly been accepted ^(70)^. Currently, the number of physicians and institutions responsible for terminal care for chronic cardiovascular diseases is limited. In addition, the course of heart failure varies among individual patients, which makes it complex. In some cases, acute care is repeatedly performed, and the course is not uniform. Because of the complexity and individuality of the clinical course of heart failure, the concept of terminal-palliative care would be different from that of other diseases, such as malignant diseases or senility deaths. It is recommended to start ACP from the early stage of heart failure. Although this concept may not adapt to Japanese culture, we should promote society-wide discussion on this issue to protect patients’ dignity.

Furthermore, in clinical practice, there are methodological difficulties in palliative care for heart failure patients in whom repeated dyspnea or systemic weakness leads to a fatal outcome.

## 7. Comprehensive Strategies to Prolong Health Expectancy

Comprehensive strategies, as described above, are necessary to reduce elderly persons’ disease burdens associated with lifestyle-related diseases and prolong health expectancy (Table 3). However, lifestyle-related diseases, including heart failure, can be prevented. To prevent such diseases in young people and disease-free persons, school education and educational activities for citizens are necessary. Lectures open to the public have been held by medical associations and scientific societies, but the government and local governments should promote continuous, national preventive education starting from compulsory education.

**Table 3.** Resources/Factors Necessary for Future Heart Failure Control.

Cooperation path
Patient notebook
Multi-occupational conference before discharge
Medical information sharing among healthcare professionals (multi-occupational conference)
Medical information sharing by regional core hospitals/home doctors (study meeting)
Joint ownership/Cloud utilization for electronic charts
Telemedicine devices
Regional rehabilitation center
Basic law on disease control

Acute-phase interventional treatment markedly improves the prognosis of patients with acute heart disease. The education of the Japanese people to promote emergency consultations, the regional establishment of an emergency transport system, arrangement of medical institution networks, and introduction of early interventional treatment through the utilization of telemedicine in medically underpopulated areas are effective. Once a disease develops, QOL improvement and secondary prevention by manifold intervention and regional integrated care, as described above, may be necessary in addition to appropriate treatment.

The economic impact of the implementation of the above-mentioned integrated healthcare system is not clear. There are a limited number of literatures regarding cost and cost-effectiveness of exercise training. Two studies indicated exercise-based rehabilitation to be a potentially cost-effective use of resources in terms of gain in quality-adjusted life year ^(50)^. Data regarding the cost-effectiveness of cardiac rehabilitation in Japan are not available, although a retrospective multicenter cohort study is currently underway. Also, the economic impact and cost-effectiveness of integrated healthcare for heart failure has not been reported. Because of differences in health care and the medical expense payment system in each country, effectiveness and social impact of medical system reform should be considered and investigated in each country from multiple aspects.

In addition, to promote these medical services, the actual status of lifestyle-related diseases, including heart failure, must be evaluated. A survey regarding the actual status and patient registration are required. To achieve such a comprehensive strategy, the national and local government’s support is necessary. In 2018, a national basic law on the countermeasures against cardiovascular diseases for this purpose has been established as a big advance in this field.

## Conclusion

With the coming of the “heart failure pandemic” era, geriatric heart failure control must be promptly established. Information sharing, social recognition of problems, new social system establishment, and support from the government are necessary. If there is no paradigm shift regarding medical systems, current medical systems in Japan may not continue to exist eventually. It is obligatory for all health care professionals and Japanese people to overcome the serious situation with wisdom.

In addition, “heart failure pandemic” will also surge in Asian countries, where the rapid aging of society is progressing, as indicated for Japan. To overcome this problem from a global viewpoint, information sharing and cooperation against the common enemy are necessary.

## Article Information

### Conflicts of Interest

None

### Sources of Funding

This work was supported by Research Grants from the Japan Agency for Medical Research and Development (AMED) grant number 16ek0210058h0001 and the Ministry of Health, Labor and Welfare, Government of Japan grant number 18062589.

### Disclaimer

Mitsuaki Isobe is one of the Associate Editors of JMA Journal and on the journal's Editorial Staff. He was not involved in the editorial evaluation or decision to accept this article for publication at all.
